# FKBP51 in the Oval Bed Nucleus of the Stria Terminalis Regulates Anxiety-Like Behavior

**DOI:** 10.1523/ENEURO.0425-21.2021

**Published:** 2021-12-17

**Authors:** Clara Engelhardt, Fiona Tang, Radwa Elkhateib, Joeri Bordes, Lea Maria Brix, Lotte van Doeselaar, Alexander S. Häusl, Max L. Pöhlmann, Karla Schraut, Huanqing Yang, Alon Chen, Jan M. Deussing, Mathias V. Schmidt

**Affiliations:** 1 Research Group Neurobiology of Stress Resilience, Max Planck Institute of Psychiatry, Munich 80804, Germany; 2 International Max Planck Research School for Translational Psychiatry (IMPRS-TP), Munich 80804, Germany; 3 Department of Stress Neurobiology and Neurogenetics, Max Planck Institute of Psychiatry, Munich 80804, Germany; 4 Department of Neurobiology, Weizmann Institute of Science, Rehovot 76100, Israel; 5 Research Group Molecular Neurogenetics, Max Planck Institute of Psychiatry, Munich 80804, Germany

**Keywords:** anxiety, BNST, FKBP5, mouse, stress

## Abstract

The cochaperone FKBP51, encoded by the *Fkbp5* gene, has been identified as central risk factor for anxiety-related disorders and stress system dysregulation. In the brain, the oval bed nucleus of the stria terminalis (ovBNST) has been implicated in stress-induced anxiety. However, the role of *Fkbp5* in the ovBNST and its impact on anxiety-like behavior have remained unknown. Here, we show in mice that *Fkbp5* in the ovBNST is reactive to acute stress and coexpressed with the stress-regulated neuropeptides *Tac2* and *Crh*. Subsequently, results obtained from viral-mediated manipulation indicate that *Fkbp5* overexpression (OE) in the ovBNST results in an anxiolytic-like tendency regarding behavior and endocrinology, whereas a *Fkbp5* knock-out (KO) exposed a clear anxiogenic phenotype, indicating that native ovBNST expression and regulation is necessary for normal anxiety-related behavior. Notably, our data suggests that a stress-induced increase of *Fkbp5* in the ovBNST may in fact have a protective role, leading to a transient decrease in anxiety and suppression of a future stress-induced hypothalamic-pituitary-adrenal (HPA) axis activation. Together, our findings provide a first insight into the previously unknown relationship and effects of *Fkbp5* and the ovBNST on anxiety-like behavior and HPA axis functioning.

## Visual Abstract

**Figure:**
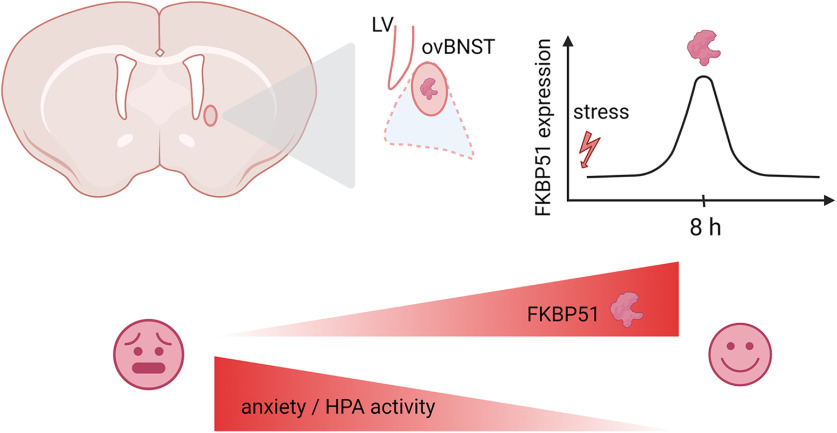

## Significance Statement

The cochaperone FKBP51 is a known risk factor for psychiatric disorders and stress system dysregulation. Both FKBP51 and the oval bed nucleus of the stria terminalis (ovBNST) have been implicated in anxiety, yet their combined effects of mediating anxiety-like states have not been explored. Here, we provide a characterization of the role of *Fkbp5* in the ovBNST on HPA axis function and anxiety-related behavior. Our findings suggest that stress induction of *Fkbp5* in the ovBNST may have a protective role, leading to decreased anxiety and suppression of a future stress-induced HPA axis activation. Overall, this study constitutes a basic step toward understanding the underlying mechanisms of *Fkbp5* signaling in the ovBNST and their role in stress-induced anxiety disorders.

## Introduction

Stress exposure can trigger maladaptive behavioral responses and induce mood disorders, such as anxiety (De Kloet et al., 2005). Anxiety is characterized by nonadaptive hypervigilance and threat overestimation in uncertain situations (Sylvers et al., 2011). The FK506 binding protein 51 (FKBP51; encoded by the *Fkbp5* gene), a heat shock protein 90 kDa (Hsp90) cochaperone, is a regulator of the stress system and a risk factor for anxiety disorders (Binder et al., 2008). Together with Hsp90, FKBP51 regulates glucocorticoid receptor (GR) activity via a short negative feedback loop. This signaling pathway rapidly restores homeostasis in the hypothalamic-adrenal-pituitary (HPA) axis after exposure to stress. Altered HPA axis regulation mechanisms, specifically impaired signaling of GR, have been causally implicated in the pathogenesis of anxiety (Holsboer, 2000).

Interestingly, single nucleotide polymorphisms (SNPs) in FKBP5 have been associated with increased FKBP51 expression and GR resistance, leading to differential HPA axis activation after stress exposure (Binder, 2009). Healthy controls homozygote for the high-induction alleles show significantly slower recovery of stress-related increases in cortisol levels as well as more anxiety symptoms in the recovery phase than healthy controls with other genotypes (Ising et al., 2008). Furthermore, carriers of the same risk variant were more susceptible to anxiety when exposed to childhood maltreatment (Scheuer et al., 2016).

Rodent studies have provided further insights into the role of FKBP51 and anxiety. Traditionally, anxiety is associated with the amygdala (Tye et al., 2011; Felix-Ortiz et al., 2016). While a global knock-out (KO) of *Fkbp5* did not affect anxiety-like behavior, reducing FKBP51 in the amygdala decreased stress-induced anxiety-like behavior (Attwood et al., 2011). Likewise, pharmacological disruption of FKBP51 signaling in the amygdala demonstrated an anxiolytic effect, whereas FKBP51 overexpression (OE) enhanced anxiety-like behavior. However, anxiety was not altered by FKBP51 OE in the dorsal hippocampus of mice (Hartmann et al., 2015). These findings suggest that FKBP51 in the amygdala regulates stress-induced anxiety-like behavior and that these effects are highly region-specific.

Like the amygdala, a large body of evidence implicates the bed nucleus of the stria terminalis (BNST) in anxiety. The BNST receives information from limbic structures [e.g., amygdala, hippocampus, medial prefrontal cortex (mPFC)] and projects to autonomic and neuroendocrine systems located in hypothalamus and brainstem regions that regulate the HPA axis (Dong et al., 2001; Ch’ng et al., 2018). Clinical imaging data suggest that BNST activity is positively correlated with increased anxiety (Somerville et al., 2010; Yassa et al., 2012). The rodent literature however has presented conflicting results (Walker and Davis, 1997; Treit et al., 1998; Duvarci et al., 2009; Van Dijk et al., 2013; Luyck et al., 2017), confirming that the BNST in fact modulates anxiety, but not specifying if this structure increases or decreases anxiety. These discrepancies might be accounted for by the anatomic and neurochemical heterogeneity of the BNST. The BNST consists of 18 subnuclei, which likely regulate anxiety in different, sometimes opposing directions (Bota et al., 2012). For instance, optogenetically inhibiting the oval nucleus of the BNST (ovBNST) elicited an anxiolytic effect, whereas light-induced decreases in neuronal activity in the anterodorsal BNST were anxiogenic (Kim et al., 2013). Likewise, chronic stress and acute optogenetic activation of the ovBNST increased anxiogenic behaviors (Hu et al., 2020a). Moreover, early life stress resulted in a long-lasting activation of CRH signaling in the mouse ovBNST, leading to potential maladaptive changes in ovBNST function in adulthood (Hu et al., 2020b). The ovBNST is therefore a promising region with regard to anxiety disorders, since it is strongly involved in mediating anxiety-like behavior.

Given FKBP51’s role in stress-induced anxiety, an understanding of its function in the ovBNST is of great relevance to further decipher maladaptive anxiety as a psychiatric disease. Here, we describe FKBP51 expression and regulation in the ovBNST under basal conditions and after stress. We further explore the neuropeptide expression profile of FKB51-positive cells within the ovBNST. Finally, we delineate the effects of *Fkbp5* manipulation in the ovBNST on anxiety-like behavior and neuroendocrinology.

## Materials and Methods

### Animals and animal housing

Experiments were conducted with C57Bl/6n, Fkbp5^lox/lox^ (Häusl et al., 2021) and Fkbp5^KO^ (Tranguch et al., 2005) mice obtained from the in-house breeding facility of the Max Planck Institute of Psychiatry, Munich. All transgenic mice were kept on a C57Bl/6n background. All animals used during the experiments were male and between 8 and 12 weeks old. Initially, mice were group housed and then single-housed at least one week before the start of the experiment. Housing parameters in the holding and testing rooms were kept constant on a 12/12 h light/dark cycle, with controlled temperature (22 ± 2°C) and humidity (45 ± 10%). Food (Altromin 1324, Altromin GmbH, Germany) and water (tap water) was provided *ad libitum*. Experiments were conducted in accordance with the European Communities Council Directive 2010/63/EU. All efforts were made to minimize animal suffering during the experiments. Protocols were approved by the committee for the Care and Use of Laboratory Animals of the Government of Upper Bavaria, Germany.

### Stress paradigms

#### Acute restraint stress (ASR)

Mice were restrained manually and guided into a 50 ml falcon tube. The falcon contained holes in the top and the lid to allow for sufficient ventilation and tail movement. The ASR lasted 4 h, and animals were either killed immediately after or allowed to recover in their home cage until the time of testing.

#### Chronic social defeat stress (CSDS)

The CSDS paradigm lasted for 21 d to allow for robust and chronic stress exposure and was conducted as described previously (Wagner et al., 2012). Briefly, experimental mice were placed in the home cage of a dominant CD1 resident mouse. Interaction was permitted until the experimental mouse was attacked and defeated by the CD1 aggressor. Mice were subsequently separated by a wire mesh that prevented physical contact but maintained sensory contact for 24 h. Each day, for 21 d, the experimental mouse was paired with another unfamiliar CD1 mouse. Both control and stressed mice were handled daily during the course of the stress exposure. Care was taken that the aggressive encounter was terminated before any injuries might occur. Mice were scored daily for possible wounds and injured animals were excluded from the experiment.

#### Acute fear conditioning

Animals were habituated for 2 d by placing them into the conditioning chamber (Bioseb) for 5 min with the chamber lights switched on. On the consecutive day (D3), mice either received five tones (30 s, 9 kHz, 80 dB) paired with a foot shock (500 ms, 0.7 mA) and a 5-min intertrial, or they were exposed to the tones only. Animals remained in the shock/tone-shock context for an additional 60 s, before they were returned to their home cages. Control animals remained in their home cages at all times. Stimuli (light, tone, shock) were operated with commercially available software (Packwin V2.0; Panlab).

### Behavioral paradigms

All behavioral testing was conducted in the animal facility of the Max Planck Institute of Psychiatry, Munich. Tests were performed during the light phase between 7 A.M. and 1 P.M. to avoid potential behavioral alterations because of circadian variation of corticosterone levels. The behavioral testing was conducted in the following order: elevated plus maze (EPM), dark-light box (DALI), open field (OF), and stress response after ASR. Recording, tracking and scoring of animal behaviors was conducted using the automated video tracking system ANY-maze (ANY-maze 6.18; Stoelting Co). All tests were performed by an experienced, blinded researcher and according to established protocols.

#### EPM

The EPM was performed as previously described (Santarelli et al., 2014). Briefly, animals were placed in an elevated (50 cm) plus-shaped platform made of gray PVC, with two opposing open arms (30 × 5 × 0.5 cm) and two opposing enclosed arms (30 × 5 × 15 cm). Illumination was <10 lux in the enclosed arms and 25 lux in the open arms. Animals were placed in the center zone facing one of both closed arms at the beginning of a 10-min trial. An increase in open arm activity (duration and/or entries) is interpreted as a decrease in anxiety-like behavior.

#### DALI

The apparatus was comprised of a dark and protected compartment (15 × 20 × 25 cm, dimly lit under 10 lux) and a brightly illuminated compartment (30 × 20 × 25 cm, lit with 700 lux); both compartments were connected by a small opening. Mice were placed in the dark compartment, facing toward a wall and recorded for 5 min. To assess anxiety-related behavior, the time spent, number of entries and latency to first entry into the lit compartment were measured.

#### OF

Mice were placed in a corner of a 50 × 50 × 50 cm plastic arena. Fifteen-minute trials were video recorded by an overhead camera. The test was performed under low light conditions (20 lux). Parameters of interest regarding anxiety-like behavior were time spent and number of entries to the center zone. For more detailed analysis, the time total was divided into three segments of 5 min.

#### ASR

Mice were restrained for 15 min, and blood was immediately collected by a tail cut to examine response corticosterone levels. Mice were then released back into their home cage; 30 min and 60 min after the onset of the stressor, additional blood samples were again collected via the tail vein to assess corticosterone levels. Plasma was collected and stored at −20°C. Levels of plasma corticosterone were subsequently determined using a commercially available radioimmunoassay kit with ^125^I-labeled anti-corticosterone antibody [MP Biomedicals Inc.; sensitivity: 12.5 ng/ml; intraassay coefficient of variation (CV): 7%; interassay CV: 7%].

### Tissue collection and processing

Animals were anesthetized with isoflurane and killed by decapitation. Basal trunk blood was collected and processed (as described above). Brains were removed, snap frozen and stored at −80°C. Adrenals and thymus gland were removed, dissected from fat and weighed. Alternatively, mice were anesthetized with isoflurane and transcardially perfused with 0.1 m PBS followed by 4% (v/v) paraformaldehyde (PFA) fixative in PBS. Brains were rapidly removed and postfixed in PFA overnight at 4°C. The following day, postfixed brains were transferred to a cryoprotectant solution (30% sucrose in 0.1 m PBS) for two additional overnight incubations at 4°C. Brains were stored at −4°C until processed.

#### Stereotactical microinjections

Manipulation of FKBP51 was performed using adeno-associated bicistronic AAV1/2 vectors (AAV1/2-HA-CAG-FKBP5-1 and AAV1/2-CAG-null, GeneDetect; AAV1-CMV-Cre and AAV1/2-CAG-empty-IRES-EGFP, Addgene; AAV1/2-ESARE-ER^T2^CreER^T2^). Stereotactic injections were performed as described previously (Schmidt et al., 2011). Briefly, an injection volume of 0.3 μl using a glass capillary with a tip resistance of 2−3 MΩ over 6 min (0.05 μl/min) was used to deliver the virus. For bilateral microinjections into the ovBNST, mice were anesthetized with isoflurane and fixed in a stereotaxic frame to target the following coordinates relative to bregma: posterior 0.8 mm, 1.2 mm lateral, 4.3 mm ventral. After surgery, mice remained in their home cage for three weeks until the start of behavioral experiments. Successful virus expression was verified by *in situ* hybridization (ISH).

### Administration of AAV-ESARE-ER^T2^CreER^T2^ and hydroxytamoxifen (4OHT) treatment

To obtain a KO of *Fkbp5* in *Fkbp5*-positive neurons of the ovBNST that had been previously activated by ASR, we used an AAV-ESARE-ER^T2^CreER^T2^ in Fkbp5^lox/lox^ mice. Animals received bilateral microinjections and three weeks after the virus injection Fkbp5^lox/lox^ mice were subjected to a 4 h restraint. To induce activity-dependent viral Cre expression, 4OHT (H6278, Sigma-Aldrich) 50 mg/ml 4OHT dissolved in DMSO (D8148, Sigma-Aldrich) and diluted 10× in saline containing 2% Tween 80 (P1754, Sigma-Aldrich) and 10× in saline; final concentration: 2.5 mg/ml 4OHT, 5% DMSO and 1% Tween 80 in saline) was injected immediately before the restraint (final dose: 25 mg/kg). Since in Fkbp5^lox/lox^ mice the exon 9 of the *Fkbp5* gene is flanked by two lox P sites, the expression of Cre recombinase, induced by the synthetic activity-dependent promoter ESARE, subsequently results in the deletion of *Fkbp5* in previously activated cells. Thus, a *FKBP5*-KO only in the neurons in the ovBNST that have been previously activated by restraint stress, is achieved. Of note: it is possible that only a robust activation of a cell will result in *Fkbp5* deletion, while a mild activation might fail to drive Cre expression. Thus, while we capture with this approach a selected subpopulation of stress-activated cells in the ovBNST, we cannot exclude a residual *Fkbp5* expression in mildly stress-activated neuronal populations.

#### Immunohistochemistry

Immunohistochemistry was used to detect β-gal distribution and thus indirectly visualize cellular location of FKBP51. In addition, virus injections were validated using immunohistochemical staining. Briefly, serial free-floating sections were washed in 0.1 m PBS and incubated in blocking solution [10% normal goat serum (NGS)/1% Triton X-100/0.1 m PBS] for 30 min at room temperature. Sections were incubated with the primary antibody (diluted 1:500−1000 in 1% NGS/0.3% Triton X-100/0.1 m PBS) overnight at 4°C. Sections were washed with 0.1 m PBS and incubated with a secondary antibody (1:500–1000) for 2 h in a light protected environment. After washes with 0.1 m PBS, the sections were mounted with DAPI medium (Fluoromount-G catalog #0100-20) and cover-slipped.

Fresh-frozen sections used for virus validation were treated with an adapted version of the protocol described above. A hydrophobic barrier PAP pen (Sigma-Aldrich) was used to encircle the sections. Blocking and antibody solutions were pipetted onto the encircled region. Furthermore, slides were covered with Parafilm (neoLab) during antibody incubation to prevent drying out and ensure constant coverage of the sections.

#### ISH

*Fkbp5* gene expression in the BNST was examined by ISH. Coronal whole-brain slices were cryosectioned at a thickness of 20 μm and directly mounted onto Superfrost Plus Slides as 6 sequential series. A cRNA antisense riboprobe was transcribed from linearized plasmid DNA (for forward primer*:* 5′CTTGGACCACGCTATGGTT; reverse primer: 5′GGATTGACTGCCAACACCTT). ISH was performed as previously described (Schmidt et al., 2003, 2007). For signal detection, slides were exposed to a Kodak Biomax MR film (Eastman Kodak Co). Exposure times varied according to the radioactive properties of each riboprobe. Autoradiographic densities were quantified using the NIH ImageJ Software to both validate and quantify expression of the gene.

#### Double ISH (DISH)

DISH was used for the colocalization of *Fkbp5* mRNA with the GABAergic marker *Gad65*/*Gad67* within the ovBNST. Briefly, ^35^S UTP-labeled *Fkbp5* riboprobe was used with a combination of digoxigenin (DIG)-labeled *Gad65* and DIG-labeled *Gad67* riboprobe. The antisense riboprobes were transcribed from a linear plasmid for *Fkbp5* (forward primer: 5′-CTT GGA CCA CGC TAT GGT TT-3′; reverse primer: 5′-GGA TTG ACT GCC AAC ACC TT-3′), *Gad65* (forward primer: 5′-TAA TAC GAC TCA CTA TAG CG-3′; reverse primer: 5′-CCC TTT AGT GAG GGT TAA TT-3′) and *Gad67* (forward primer: 5′-ATG ACG TCT CCT ACG ATA CA-3′; reverse primer: 5′-CCC CTT GAG GCT GGT AAC CA-3′). DISH was performed as previously described (Refojo et al., 2011). Briefly, sections were fixed in 4% PFA. After several washing steps, endogenous peroxidase was quenched in 1% H_2_0_2_. Background was reduced in 0.2 m HCl, followed by 2 additional washing steps (1 × PBS). The slides were then acetylated in 0.1 m triethanolamine, washed (1 × PBS) and dehydrated through increasing concentrations of ethanol. After that, tissue sections were saturated with 90 μl of hybridization buffer containing ∼50,000 cpm/μl ^35^S-labeled riboprobe and 0.2 μg/ml DIG-labeled riboprobe. Brain sections were cover-slipped and incubated overnight at 55°C. The following day, coverslips were removed and sections were washed several times in decreasing concentrations of SSC/formamide buffers under stringent temperature settings. After SSC washes, sections were treated with RNase A in 1 × NTE at 37°C and washed in 1× NTE/0.05% Tween 20 (2× times) followed by a blocking step in 4% BSA for 1 h. After additional washing steps, sections were blocked in NEN-TNB for 30 min. In a final step, slides were incubated with Roche’s anti-DIG (FAB; 1:400, Roche Molecular Diagnostics) at 4°C overnight. On the last day, sections were washed several times in TNT at 30°C followed by a signal amplification step in which sections were incubated for 15 min in tyramide-biotin. Thereafter, additional washing steps were performed (Roche washing buffer, Roche Molecular Diagnostics). Sections were then incubated for 1 h with Roche streptavidin-AP (1:400, Roche Molecular Diagnostics). Afterwards, sections were washed in Roche washing buffer and subsequently prepared for Vector red staining in 100 mm Tris/HCl (Vector Laboratories). Slides were immersed in Vector red solution under unlit conditions for 15–30 min depending on staining. When staining was sufficient, the reaction was stopped in 1 × PBS followed by a fixation step in 2.5% glutaraldehyde. Finally, sections were washed in 0.1 × SSC and dehydrated through a graded series of ethanol solutions (30%, 50%, 70%, and 96%).

#### RNAscope

RNAscope is a novel ISH assay for detection of target RNA within intact cells. This approach allows for multiplex detection of up to four target genes. The procedure was performed according to manufacturer’s specifications and with the RNAscope fluorescent Multiplex Reagent kit (catalog #320850, Advanced Cell Diagnostics). The probes used for detection were; Fkbp5 (Mm-Fkbp5-No-XHs), Tac2 (Mm-Tac2-C2/C3), Gad65 (Mm-Gad2-C3), and Crh (mm-Crh-C2). Briefly, sections were fixed in 4% PFA for 30 min at 4°C and dehydrated in increasing concentrations of ethanol. Next, tissue sections were incubated with protease IV for 30 min at room temperature. The probes were mixed at a ratio of 1:1:50 and hybridized for 2 h at 40°C followed by four hybridization steps of the amplification reagents 1–4. The sections were then counterstained with DAPI, cover-slipped and stored at 4°C. Images were acquired with experimenter blinded to probes used. Sixteen-bit images of each section were taken on a Zeiss confocal microscope using a 20×, 40×, and 63× objective. For quantification three images of each ovBNST per animal (*n* = 6 animals per condition) were taken with identical settings for laser power, detector gain, and amplifier offset. *Fkbp5*, *Tac2*, and *Crh* mRNA was counted manually and each cell containing three dots was counted as positive.

### Statistical analyses

Data were analyzed using IBM SPSS Statistics 25 software (IBM SPSS Statistics, IBM) and GraphPad Prism 8.0 (GraphPad Software). For the comparison of two groups, the independent Student’s *t* test was applied. If the data were not normally distributed, the nonparametric Mann–Whitney (MW test) was used. Data with more than two groups were tested by the appropriate ANOVA model followed by Bonferroni *post hoc* analysis to determine statistical significance between individual groups. In the event of multiple time points, a repeated-measures ANOVA was performed. If a single value was missing from the data, mixed model analysis was applied. All data are shown as means ± SEM. The level of statistical significance was set at *p *<* *0.05.

## Results

### Expression and regulation of *Fkbp5* in the ovBNST

**Figure 1.**
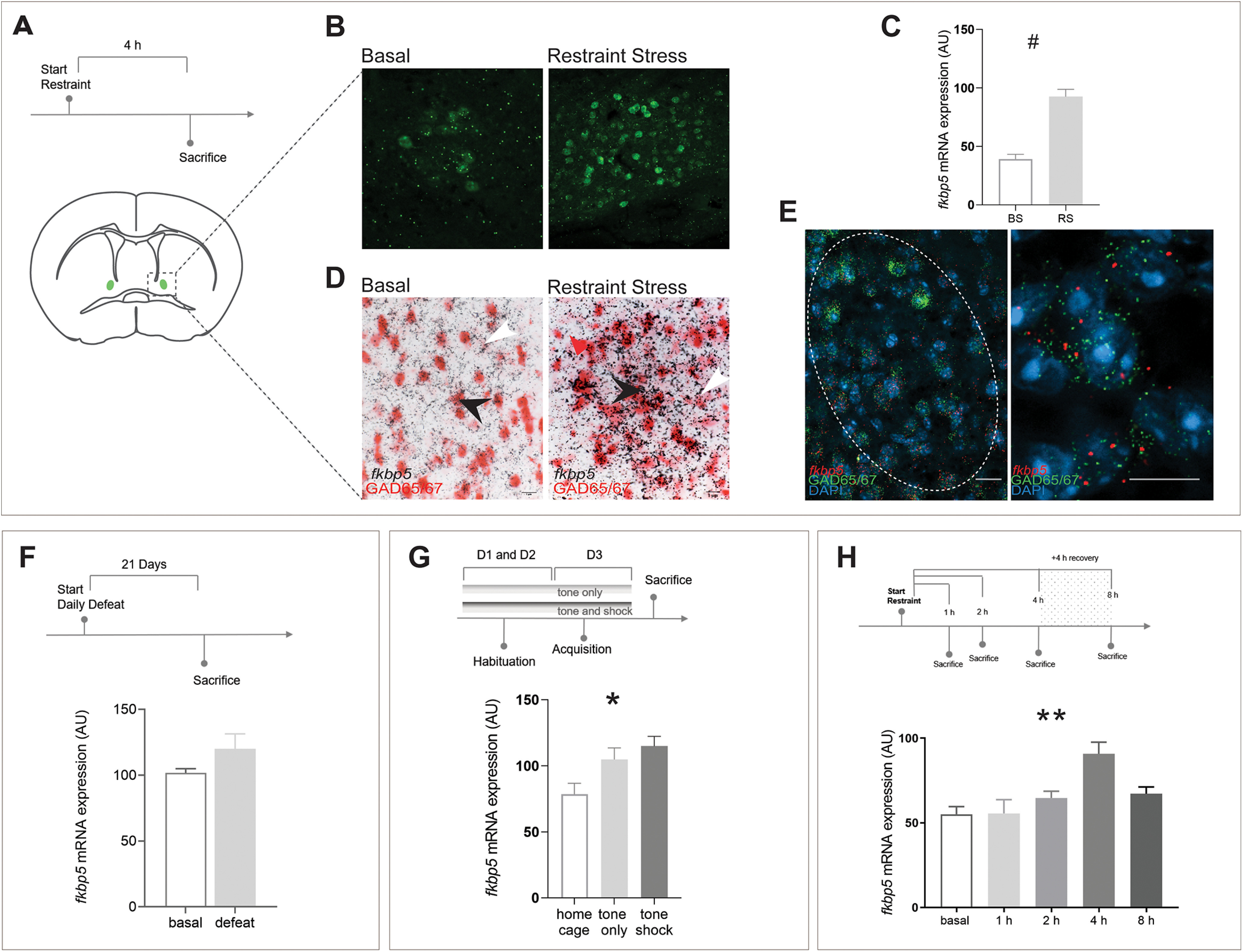
Expression and regulation of *Fkbp5* in the ovBNST. ***A***, *Fkbp5* is among other regions expressed in the ovBNST at basal level and highly upregulated after exposure to acute stress. Stress regulation of FKBP51 is shown through β-gal upregulation (***B***) and on mRNA level (***C***). Furthermore, *Fkbp5* is expressed and regulated in the majority of GABAergic neurons (***D***). Black arrow, GABAergic cell expressing *Fkbp5*; white arrow, non-GABAergic cell expressing *Fkbp5*. This was further confirmed by RNAscope (***E***), demonstrating a clear coexpression of *Fkbp5* and GAD65. ***F***, *Fkbp5* was not significantly upregulated after exposure to CSDS. However, exposure to a different type of acute stress, acute fear conditioning, reliably resulted in a significant upregulation of *Fkbp5* in the ovBNST (***G***). A time course of *Fkbp5* revealed a significant upregulation, with *Fkbp5* regulation peaking at its highest after 4 h (***H***). Data are mean ± SEM; *p < 0.05, **p < 0.01, #*p* < 0.001.

To gain a deeper understanding of the possible function of FKBP51 in the BNST, we first explored its regulation following exposure to ASR. Scharf and colleagues previously showed FKBP51 upregulation after ASR in various regions including the paraventricular nucleus of the hypothalamus (PVN) and central nucleus of the amygdala (CeA); however, the BNST has remained unexplored and therefore poses a novel region of interest (Scharf et al., 2011). Since visualization of FKBP51 protein regulation is hindered by the lack of specific antibodies, we used the Fkbp5^KO^ line. Here, the insertion of the *lacZ* gene in the *Fkbp5* locus of this line results in changes in β-gal expression, which directly reflects expression changes of FKBP51. β-Gal free-floating IHC indicated a strong upregulation of FKBP51 after exposure to ASR in the ovBNST (Fig. 1*A*,*B*). This finding was confirmed using ISH to quantify *Fkbp5* mRNA expression in C57Bl/6n mice at basal level (*n* = 7) and after exposure to an acute 4-h restraint stress. Exposure to ASR resulted in a significant increase in *Fkbp5* mRNA expression within the ovBNST (*t*_(12)_ = 6.61, *p* < 0.0001, unpaired *t* test; *n* = 8; Fig. 1*C*). To further characterize the *Fkbp5*-positive neuronal population activated by ASR within the ovBNST, coexpression with the neuronal GABAergic markers GAD65/67 was determined using DISH at basal level and after restraint stress (Fig. 1*D*). From the results it could be concluded that *Fkbp5* is expressed and regulated in the majority of GABAergic neurons in the ovBNST. This finding was further confirmed using RNAscope (Fig. 1*E*), demonstrating a clear coexpression of the marker GAD65 and *Fkbp5* within the ovBNST and on a single cellular level. Interestingly, quantification of *Fkbp5* mRNA regulation after exposure to CSDS did not result in a significant difference between the control (*n* = 10) and stressed group (*n* = 8) in the ovBNST (Fig. 1*F*), indicating that ovBNST *Fkbp5* upregulation might be reactive specifically to acute stress exposure. Consequently, mice were exposed to a different kind of acute stressor, a modified and acute version of classical fear conditioning that included a home cage only group (*n* = 7), tone only (*n* = 8), and shock and tone combined group (*n* = 5). A one-way ANOVA demonstrated a significant difference between the three groups (*F*_(2,17)_ = 4.644, *p* = 0.025; Fig. 1*G*) and Bonferroni *post hoc* analysis revealed a significant difference between the home cage group and the tone and shock combined group (*t*_(17)_ = 2.835, *p* = 0.034). Finally, to gain information on the dynamics of *Fkbp5* upregulation within the ovBNST after exposure to ASR, a time course was performed (Fig. 1*H*). *Fkbp5* upregulation differed significantly within the different time points (*F*_(4,21)_ = 5.767, *p* = 0.0027, ANOVA) and peaked as observed previously after 4 h (Bonferroni basal vs 4 h, *t*_(21)_ = 3.848, *p* = 0.0093).

### Coexpression of *Fkbp5* with Crh and Tac2

**Figure 2.**
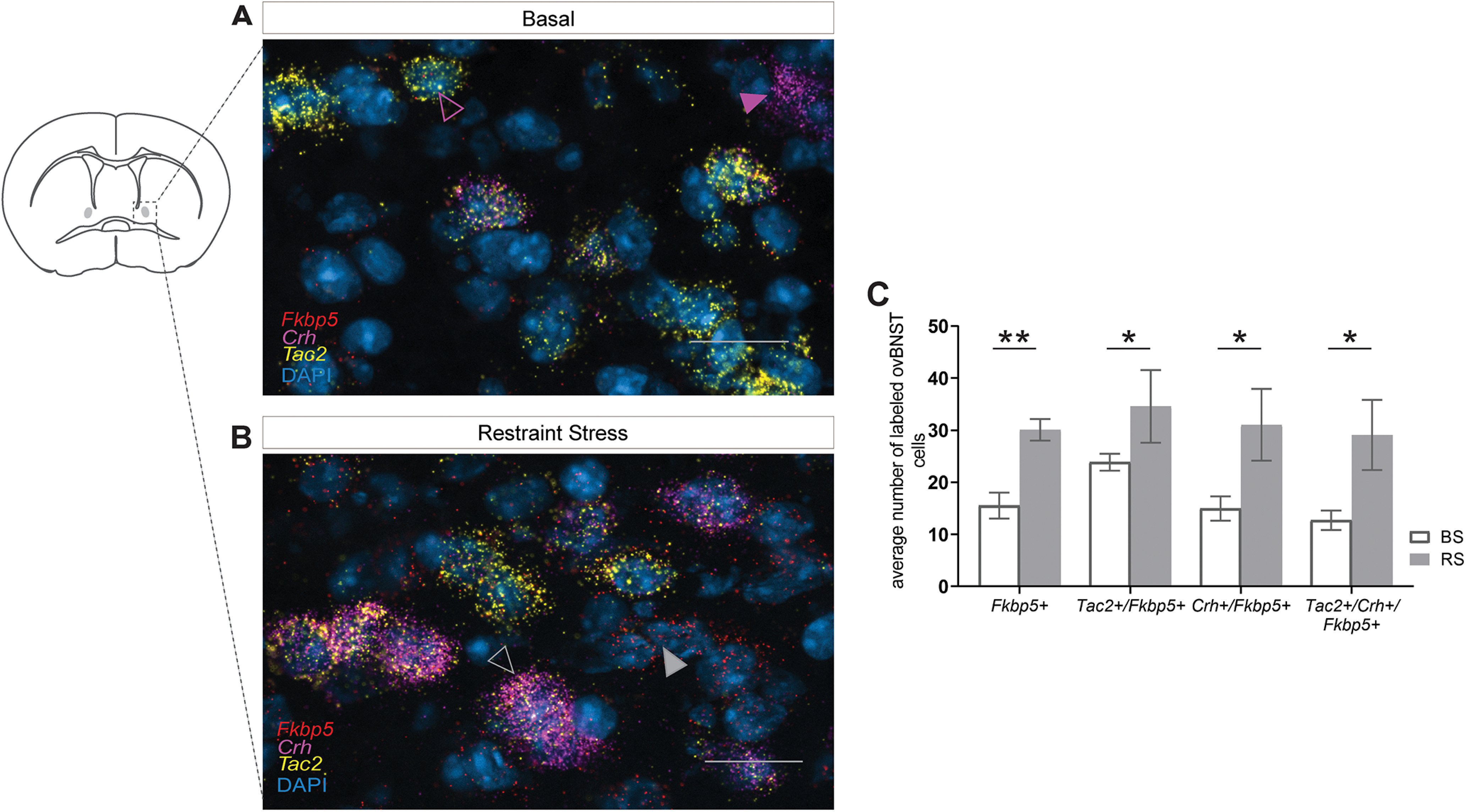
*Fkbp5+* cells in the ovBNST coexpress with the neuropeptides *Crh* and *Tac2* and their number is significantly increased after exposure to ASR. ***A***, *Fkbp5* and *Tac2* are coexpressed in the ovBNST, as can be seen in detail (violet outline arrow). *Fkbp5* and *Crh* are also coexpressed in the ovBNST as shown in detail (violet arrow). ***B***, Expression patterns of *Fkbp5* with *Tac2* and *Crh* in the ovBNST also strongly overlapped (gray outline arrow). In addition, there were some cells that expressed *Fkbp5* only (gray arrow). ***C***, Quantification of the number of cells expressing *Fkbp5* only, coexpressing *Fkbp5* and *Tac2*, coexpressing *Fkbp5* and *Crh*, and coexpressing *Fkbp5*, *Tac2*, and *Crh* after exposure to ASR resulted in significant upregulation across all cell types. Scale bar (***A***, ***B***): 25 μm. Data are mean ± SEM; *p < 0.05, **p < 0.01.

Next, we wanted to explore the neurochemical profile of *Fkbp5*-positive neurons in the ovBNST. Previous studies have demonstrated that various stress-related and anxiety-related neuropeptides are richly expressed in the BNST (Walter et al., 1991; Adhikari, 2014; Adhikari et al., 2015; Zelikowsky et al., 2018; Hu et al., 2020a). In fact, the highest concentration of Crh neurons in the brain is located in the ovBNST (Morin et al., 1999; Daniel and Rainnie, 2016). Another neuropeptide of interest that has been recently associated with the BNST and stress is Tachykinin 2 (*Tac2*; Zelikowsky et al., 2018). Thus, here we investigated the potential coexpression of *Fkbp5* with *Crh* and *Tac2* in the ovBNST (Fig. 2). Moreover, we explored whether coexpression levels would be altered after exposure to ASR. Our results indicate that *Fkbp5* and *Tac2* are coexpressed in the ovBNST; this also applies to *Fkbp5* and *Crh*, which are similarly coexpressed in ovBNST cells (Fig. 2*A*,*C*). Apart from these two distinct populations, coexpression patterns of *Fkbp5*, *Tac2*, and *Crh* also heavily overlapped within the ovBNST (Fig. 2*B*,*C*). Both the number of *Fkbp5*-positive cells coexpressing *Tac2* (*U* = 0, *p* = 0.002 MW test) or *Crh* (*t*_(10)_ = 2.6, *p* = 0.026, unpaired *t* test) were significantly upregulated after exposure to ASR. Most importantly, however, this was also observed in the number of *Fkbp5*-positive cells expressing both *Tac2* and *Crh* simultaneously (*t*_(10)_ = 2.43, *p* = 0.034, unpaired *t* test). We visualized *Fkbp5*-positive cells coexpressing *Tac2* (Fig. 2*A*, violet outline arrow), *Fkbp5*-positive cells strongly coexpressing *Crh* (Fig. 2*A*, violet arrow), cells expressing *Fkbp5* only (Fig. 2*B*, gray arrow), and *Fkbp5*-positive cells strongly coexpressing both *Tac2* and *Crh* (Fig. 2*B*, gray outline arrow) by using RNAscope.

### OE of *Fkbp5* in the ovBNST

**Figure 3.**
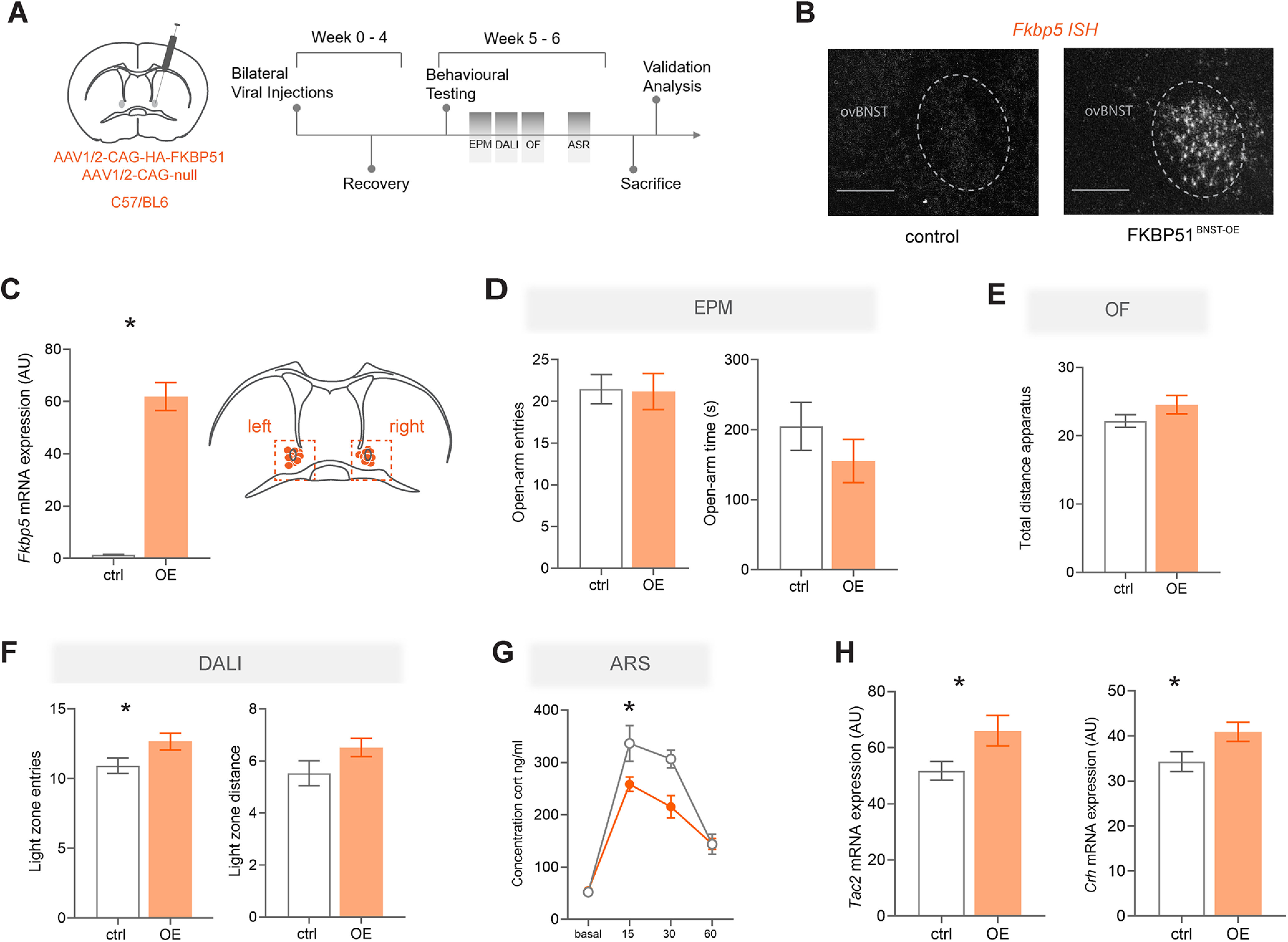
OE of *Fkbp5* in the BNST. ***A***, Schematic representation of viral manipulation and experimental timeline including testing battery. ***B***, *Fkbp5* ISH demonstrating correct viral expression (scale bar: 250 μm). ***C***, Quantification of *Fkbp5* OE. The right panel illustrates the virus injection sites. ***D***, There were no significant differences in open-arm time spent and number of open-arm entries in the EPM. ***E***, FKBP51^BNST-OE^ and control animals also did not differ in the total distance covered during the OF test. ***F***, FKBP51^BNST-OE^ animals entered the lit compartment of the DALI more frequently than control animals, but there was no significant difference in the distance covered within the light zone. ***G***, FKBP51^BNST-OE^ animals demonstrated significantly lower corticosterone levels after a 15-min ARS. ***H***, Both the coexpressed neuropeptides *Tac2* and *Crh* were significantly upregulated in FKBP51^BNST-OE^ animals. Data are mean ± SEM; *p < 0.05, **p < 0.01.

Following the characterization of *Fkbp5* expression and regulation after exposure to stress within the ovBNST, we investigated whether manipulation of *Fkbp5* would result in a behavioral phenotype and affect endocrinological parameters. Thus, viral-mediated gene transfer to the ovBNST was used to overexpress FKBP51, thereby mimicking a stress-related upregulation (Fig. 3*A*). ISH of coronal sections was used to validate *Fkbp5*-OE (*t*_(12)_ = 5.766, *p* < 0.0001, unpaired *t* test) and to exclude mice who presented off-target injection sites (Fig. 3*B*,*C*). In all mice, robust OE of *Fkbp5* was achieved within the ovBNST and adjacent dorsal BNST regions. Mice that were not infected bilaterally in the ovBNST were excluded from all analyses.

In total, 13 control and 13 FKBP51^BNST-OE^ mice were used for further analysis. There were no significant differences between control and FKBP51^BNST-OE^ animals in the time spent and the number of entries to the open arms in the EPM (Fig. 3*D*). In addition, FKBP51^BNST-OE^ and control animals did not differ in the total distance covered during the OF test (Fig. 3*E*). However, FKBP51^BNST-OE^ animals entered the lit zone of the DALI test significantly more often than control animals (*t*_(23)_ = 2.114, *p* = 0.046, unpaired *t* test), yet did not significantly differ in the distance covered within the light department (Fig. 3*F*). Furthermore, FKBP51^BNST-OE^ animals demonstrated significantly lower corticosterone levels after a 15-min ASR (*F*_(3,68)_ = 4.310, *p* = 0.008, ANOVA; Fig. 3*G*). Interestingly, subsequent ISH analysis of the coexpressed neuropeptides *Tac2* and *Chr* in FKBP51^BNST-OE^ animals revealed a significant increase in mRNA levels for both *Tac2* (*t*_(20)_ = 2.315, *p* = 0.031, unpaired *t* test) and *Crh* (*t*_(18)_ = 2.143, *p* = 0.046, unpaired *t* test), respectively (Fig. 3*H*). While the experiment overall did not yield a clear behavioral phenotype, it alluded to the idea that *Fkbp5*-OE might have a mild anxiolytic-like effect and suppress HPA axis reactivity.

### KO of *Fkbp5* in the ovBNST

**Figure 4.**
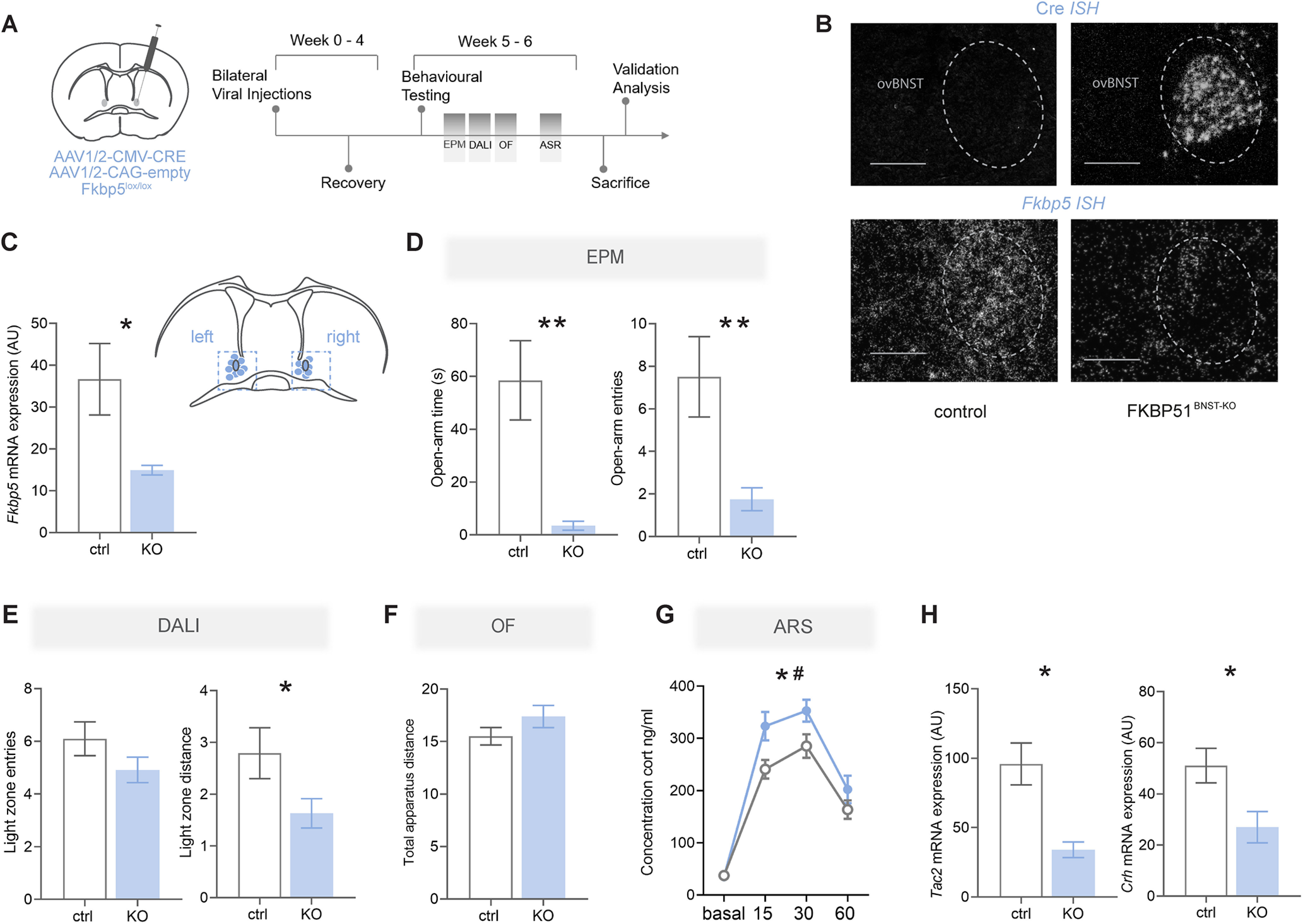
KO of *Fkbp5* in the BNST. ***A***, Schematic of viral manipulation as well as experimental timeline and testing battery. ***B***, Example of virus expression and correct targeting through *Cre* and *Fkp5 ISH*. Scale bar: 250 μm. ***C***, Quantification of *Fkbp5* knock-down in the ovBNST. The right panel illustrates the virus injection sites. ***D***, In the EPM, FKBP51^BNST-KO^ animals (*n* = 13) spent significantly less time in the open arms and entered open arms less frequently than control animals (*n* = 10). ***E***, FKBP51^BNST-KO^ animals covered significantly less distance within the light area of the dark-light box (DALI) test. ***F***, Total distance covered within the OF test was not significantly different. ***G***, FKBP51^BNST-KO^ animals demonstrated a matching neuroendocrine phenotype, exposing significantly higher corticosterone levels after a 15-min ASR. ***H***, In line with previous results, the coexpressed neuropeptides *Tac2* and *Crh* were significantly downregulated in FKBP51^BNST-KO^ animals. Data are mean ± SEM; *p < 0.05, **p < 0.01, #*p* < 0.001.

The next experiment therefore explored the necessity of FKBP51 in the ovBNST on anxiety-related behavior and HPA axis regulation by specific *Fkbp5* deletion. *Fkbp5*-KO in the ovBNST was induced by viral manipulation and animals underwent the exact same testing battery as in the previous experiment (Fig. 4*A*). *Cre* and *Fkbp5* ISH (*t*_(5)_ = 2.986, *p* = 0.031, unpaired *t* test) were used to validate *Fkbp5*-KO and correct targeting of the ovBNST (Fig. 4*B*,*C*), without excluding additional targeting of close-by regions in the dorsal BNST. Mice that were not infected bilaterally in the ovBNST were excluded from all analyses.

Interestingly, FKBP51^BNST-KO^ animals spent significantly less time in the open arms (*U* = 22, *p* = 0.001, MW test) and entered open arms of the EPM significantly less than control animals (*t*_(20)_ = 3.171, *p* = 0.005, unpaired *t* test; Fig. 4*D*). Moreover, while there was no significant difference for the number of entries into the light zone of the DALI test, FKBP51^BNST-KO^ animals did cover significantly less distance within the light zone (*t*_(19)_ = 2.097, *p* = 0.049,unpaired *t* test; Fig. 4*E*). However, the two groups did not differ in the total distance covered during the OF test, indicative of unaltered locomotor behavior (Fig. 4*F*). Furthermore, FKBP51^BNST-KO^ animals exposed significantly higher corticosterone levels after a 15-min ASR (time: *F*_(2.895,60.80)_ = 111.1, *p* < 0.0001; condition: *F*_(1,21)_ = 5.308, *p* = 0.032; Fig. 4*G*). In line with previous results, mRNA levels of *Tac2* (*t*_(5)_ = 4.34, *p* = 0.007, unpaired *t* test) and *Crh* (*t*_(5)_ = 2. 63, *p* = 0.047, unpaired *t* test) were significantly reduced in FKBP51^BNST-KO^ animals (Fig. 4*H*). Overall, these data confirm that *Fkbp5* deletion in the ovBNST has a specific effect on stress-induced anxiety and highlight an anxiogenic phenotype for *Fkbp5-*KO.

### Activity-dependent KO of *Fkbp5* in Fkbp5+ neurons of the ovBNST

**Figure 5.**
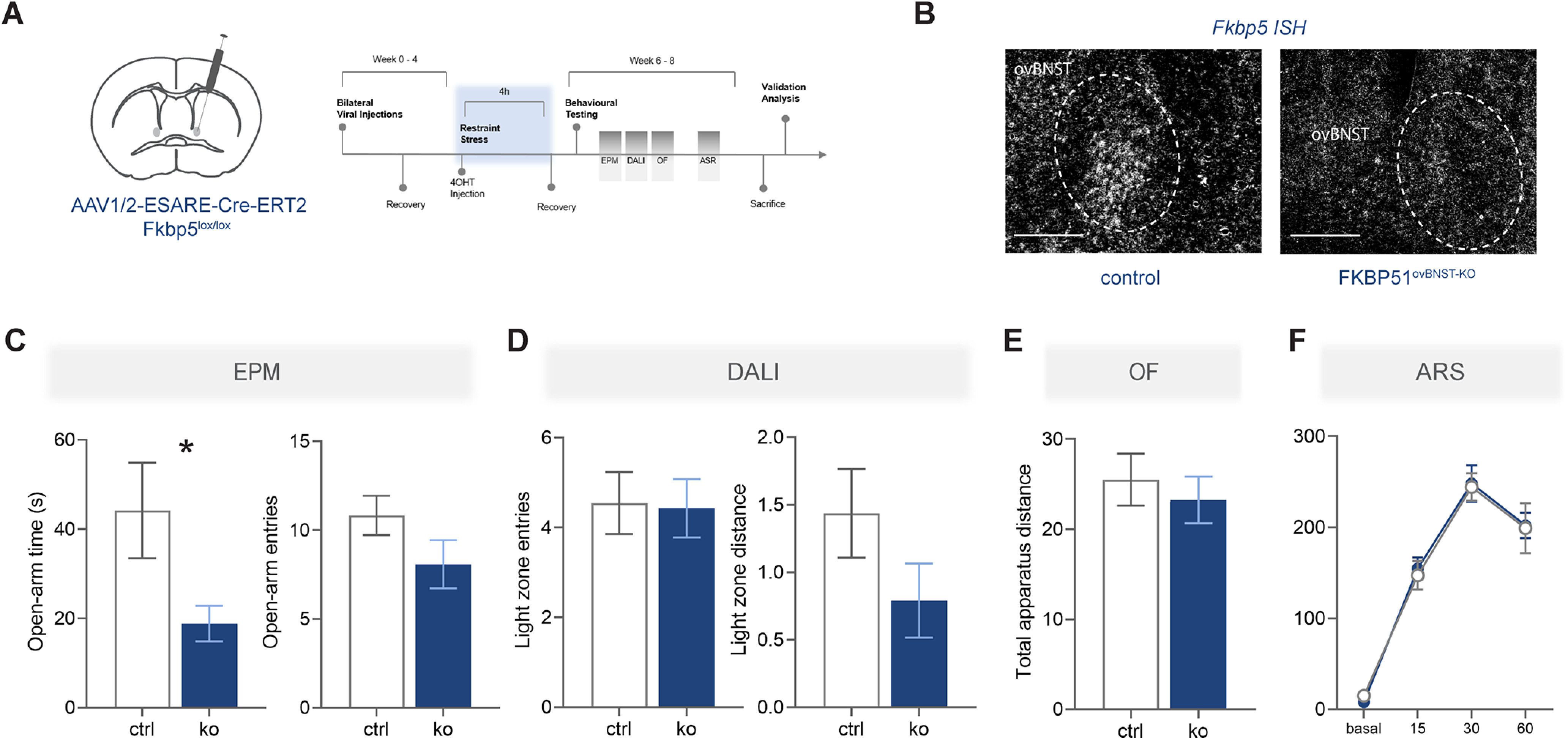
Activity-dependent KO of *Fkbp5* in the BNST. ***A***, Schematic representation of ESARE promoter driven and 4OHT-dependent conditional KO of previously activated neurons in the ovBNST, as well as subsequent experimental timeline. ***B***, *Fkbp5* ISH to validate correct viral manipulation. Scale bar: 250 μm (***C–F***). Activity-dependent KO of *Fkpb5* in the ovBNST indicated an anxiogenic phenotype. ***C***, FKBP51^ovBNST-KO^ (KO) animals spent significantly less time in the open arms of the EPM. In addition, they showed a tendency to travel less distance within the light zone of the dark-light box (DALI). The OF test (***E***) and the ASR response (***F***) did not show any significant differences between the two groups. Data are mean ± SEM; *p < 0.05, **p < 0.01.

Next, we investigated whether the lack of FKBP51 only in stress-activated cells of the ovBNST can recapitulate the anxiogenic phenotype observed in the KO experiment. Thus, *Fkbp5* was deleted in *Fkbp5*-positive neurons that had been previously activated by ASR (Fig. 5*A*). Validation of correct targeting and KO was performed through *Fkbp5* ISH (Fig. 5*B*). As in previous experiments, animals that were not infected bilaterally were excluded from all analysis.

Similar to the *Fkbp5-*KO in the BNST, activity-dependent KO of *Fkbp5* in the ovBNST indicated an anxiogenic phenotype. FKBP51^ovBNST-KO^ animals spent significantly less time in the open arms (*t*_(21)_ = 2.142, *p* = 0.044, unpaired *t* test), but did not differ to the control group regarding the number of entries to the open arms (Fig. 5*C*). Furthermore, there was no significant difference in light zone entries or distance traveled during the DALI box (Fig. 5*D*). This was also observed for the total distance traveled in the OF test (Fig. 5*E*). Finally, there was no effect of *Fkbp5* ovBNST KO on endocrinological parameters following ASR (Fig. 5*F*). Thus, a more precise KO of *Fkbp5* in the ovBNST demonstrated an anxiogenic phenotype specific to the exploration of the unprotected arms of the EPM, aligning with the previous anxiety phenotype of *Fkbp5-*KO in the ovBNST.

### Anxiolytic-like behavior after exposure to ASR

**Figure 6.**
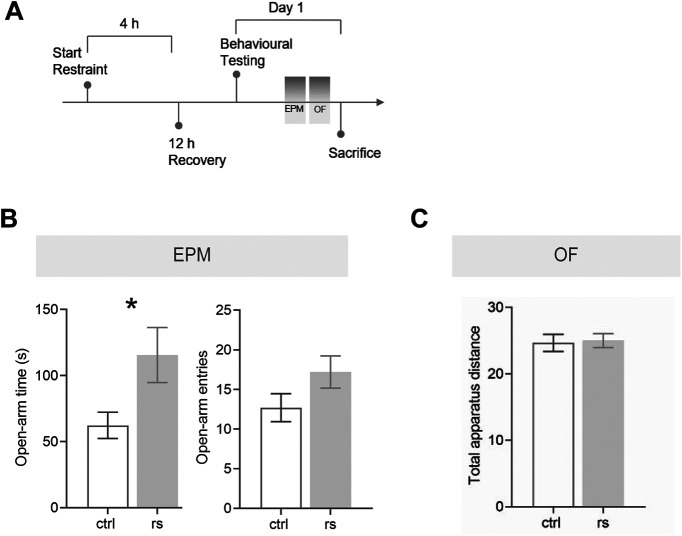
Timeframe of anxiety-like behavior after exposure to ASR. Exposure to ASR caused an anxiolytic-like phenotype. ***A***, Schematic representation of experimental timeline. ***B***, In the EPM, restrained animals (rs) spent significantly more time in the open arms. However, there was no difference regarding the number of entries to the open arms between the two groups. ***C***, The OF test did not show any significant differences between the two groups. Data are mean ± SEM; *p < 0.05, **p < 0.01.

The data on FKBP51 regulation in the ovBNST and anxiety-related behavior suggest that stress-induced increase of FKBP51 in this region might in fact have a protective role, thus leading to decreased anxiety. Consequently, we hypothesized that acute stress exposure should have a transient anxiolytic-like effect following the stress-induced FKBP51 upregulation within the ovBNST. To explore this hypothesis, C57Bl/6n mice were subjected to the EPM and OF test 12 h after exposure to the ASR paradigm (Fig. 6*A*). The time point of 12 h after stress onset was selected to allow increased FKBP51 protein expression and subsequent FKBP51-mediated effects on GR signaling. Notably, animals that had been previously restrained spent increased time in the open arms of the EPM (*t*_(18)_ = 2.306, *p* = 0.033, unpaired *t* test) compared with controls (Fig. 6*B*). Furthermore, there were no differences in total distance traveled within the OF apparatus test (Fig. 6*C*). These findings suggest that indeed acute stress exposure might lead to an anxiolytic-like phenotype because of the specific increase of FKBP51 in the ovBNST.

## Discussion

FKBP51 and the ovBNST play a pivotal role in stress-induced anxiety disorders (Ising et al., 2008; Kang et al., 2012; Kim et al., 2013; Klengel et al., 2013; Scheuer et al., 2016; Hu et al., 2020a,b). However, the precise function of FKBP51 in the ovBNST and its role in anxiety remained unknown. Here, we explore for the first time the function of FKBP51 in the ovBNST on stress, and the subsequent effects on maladaptive anxiety behaviors, revealing ovBNST FKBP51’s important role in anxiety. We demonstrate that ovBNST *Fkbp5* expression is upregulated after exposure to acute stress and coexpressed with relevant stress-related neuropeptides. Furthermore, manipulation of *Fkbp5* in the BNST has an effect on anxiety-like behaviors. Remarkably, our results indicate that stress-induced increase of *Fkbp5* in that region might have a protective role regarding anxiety-like behavior.

### *Fkbp5* expression and regulation is highly region and cell type specific

Exposure to ASR significantly upregulated *Fkbp5* in the ovBNST. This upregulation seems reactive to acute stress only, since exposure to CSDS did not result in a significant upregulation. Another acute stressor confirmed this, showing *Fkbp5* upregulation relative to stressor severity. As the ovBNST is a predominantly GABAergic nucleus (Lebow and Chen, 2016), we confirmed *Fkbp5* expression and regulation in the majority of ovBNST GABAergic neurons, which reaches a maximum at around 4 h after stress onset. The short-lasting but robust upregulation of ovBNST *Fkbp5* suggested a functional consequence of this regulation in the aftermath of an acute or traumatic stress exposure.

Furthermore, the ovBNST expresses a number of neuropeptides relevant for emotional regulation (Hammack et al., 2009; Lebow and Chen, 2016; Hu et al., 2020a), with CRH linked to anxiety-like states in the BNST (Butler et al., 2016; Faria et al., 2016; Pomrenze et al., 2019; Hu et al., 2020a). CRH is most strongly expressed in the ovBNST and responsive to stress exposure (Dabrowska et al., 2013). Similarly, neurokinin B (encoded by *Tac2*) was recently related to the BNST, stress and anxiety-like behavior. Studies of *Tac2* in the central amygdala have previously implicated the peptide in fear memory and learning (Andero et al., 2014, 2016). Zelikowsky and colleagues demonstrated that social isolation stress resulted in a robust increase of *Tac2* mRNA expression in the CeA and the anterior dorsal BNST, linking *Tac2* expression to an anxiogenic phenotype (Zelikowsky et al., 2018). We therefore assessed potential coexpression of *Fkbp5* with *CRH* and *Tac*2. Our results indicated that *Fkbp5*-positive neurons in the ovBNST coexpress *Tac2* and *Crh*. Interestingly, expression patterns of *Fkbp5* with *Crh* and *Tac2* also strongly overlapped and the significant increase of *Fkbp5/Tac2/Crh*-positive cells in the ovBNST after exposure to ASR suggest that these two neuropeptides might work in tandem with *Fkbp5* to mediate anxiety-like behavior. This was further underlined by the distinct upregulation of *Tac2* and *Crh* in FKBP51^BNST-OE^ animals and, vice versa, downregulation of the two neuropeptides in FKBP51^BNST-KO^ animals.

### Tonic *Fkbp5* expression in the ovBNST shapes HPA axis responsivity to acute challenges

The anterior BNST acts as relay station between the mPFC and the PVN, mediating HPA responses to stress to activate the HPA axis to release corticosterone (Radley and Sawchenko, 2011; Lebow and Chen, 2016). The ovBNST expresses neuropeptides that innervate CRH-expressing neurons, and stimulate activation of the HPA axis (Hammack et al., 2009; Roman et al., 2014). Here, we observed reduced or suppressed HPA axis activity as a result of long-term ovBNST *Fkbp5*-OE and enhanced HPA axis response because of stable ovBNST *Fkbp5-*KO. Since there was no effect on HPA axis responsivity to acute stress when *Fkbp5-*KO was exclusively in ovBNST stress-activated neurons, we assume, however, that the long-term HPA axis impact of *Fkbp5* manipulation is not carried by the stress-activated neuronal population of the ovBNST, but possibly by a different distinct cellular population within this nucleus (Hammack et al., 2009; Roman et al., 2014). These findings point toward differential long-lasting changes in the responsivity of the HPA axis that potentially depend on *Fkbp5* status in the BNST.

The HPA response might be different in the immediate aftermath of a stressor that leads to transient ovBNST *Fkbp5* upregulation. Homotypic stress often results in habituation of the HPA axis whereas heterotypic stress can result in its sensitization (Kirschbaum et al., 1995; McEwen, 2003; Wüst et al., 2005; Gianferante et al., 2014). However, these processes are highly time-dependent, as a blunted HPA axis response was previously observed if stressors were applied within 90 min (De Souza and Van Loon, 1982), but not after 150 min (Dallman and Jones, 1973) of the initial stress exposure. Recently, GR signaling in CRH neurons of the PVN has been implicated in long-term stress habituation (Dournes et al.,2020). Taken together, our data suggest that ovBNST *Fkbp5* likely plays a key role in adaptive HPA axis responses to repeated stressors.

### Native ovBNST *Fkbp5* expression and regulation is necessary for normal anxiety-related behavior

Our genetic manipulation of native *Fkbp5* expression in the ovBNST demonstrates the central role of this cochaperone in regulating anxiety-related behavior. While a stable and long-lasting *Fkbp5*-OE only led to a modest anxiolytic-like phenotype, possibly because of ectopic expression of *Fkbp5* in adjacent dorsal BNST structures, a deletion of *Fkbp5* in the ovBNST or specifically in stress-activated cells of the ovBNST resulted in anxiogenesis. These findings are in contrast to most of the findings in other brain regions, where virally-mediated *Fkbp5-*OE resulted in an anxiogenic phenotype (Attwood et al., 2011; Hartmann et al., 2015; Criado-Marrero et al., 2019), whereas *Fkbp5-*KO or pharmacological inhibition were associated with more resilient behavior (Hartmann et al., 2015; Volk et al., 2016). However, these previous results were reported mainly from *Fkbp5* manipulations in the amygdala (Attwood et al., 2011; Hartmann et al., 2015; Volk et al., 2016; Criado-Marrero et al., 2019). Manipulations of *Fkbp5* in other regions, such as the dorsal hippocampus of mice, did not alter anxiety-like behavior (Hartmann et al., 2015). In addition, different BNST subregions are known to regulate anxiety in opposite directions (Kim et al., 2013), and there is likely even a functional differentiation among cells located in the same BNST subnucleus (Jennings et al., 2013). Thus, the ovBNST seems to play a special role in the extended amygdala network and *Fkbp5* in this region may act to reduce stress-induced anxiety. This might also explain the lack of anxiety-related phenotypes in the full *Fkbp5-*KO mice (Hartmann et al., 2012), where *Fkbp5* is neither expressed in the ovBNST nor in the amygdala.

### Stress-induced *Fkbp5* in the ovBNST may control transient stress-related anxiolysis

Our data suggest that the stress-induced increase of *Fkbp5* in the ovBNST might be protective, leading to temporary anxiolysis. While this might seem counterintuitive given the reported anxiogenesis following stress exposure (MacNeil et al., 1997; Korte et al., 1999; Chotiwat and Harris, 2006; Grillon et al., 2007), anxiolysis has been reported before (Radulovic et al., 1998; Laxmi et al., 2003; Albrecht et al., 2013). Specifically, anxiolytic behavior in the EPM was shown after highly aversive context conditioning (Radulovic et al., 1998; Laxmi et al., 2003) and after single fear conditioning (Albrecht et al., 2013). Here, the observation of anxiolysis was always time-restricted and followed a delay after the stress exposure, possibly matching the time course of *Fkbp5* expression in the ovBNST. This hypothesis is supported by our data showing that a single episode of restraint stress leads to reduced anxiety 12 h later.

Interestingly, the phenomenon of alleged anxiolytic or resilient behavior after a stressful or traumatic event has also been reported clinically. Moreover, the BNST has been repeatedly associated with PTSD-like-behavior (Buff et al., 2017; Awasthi et al., 2020). Several studies have identified exposure to traumatic events and subsequent symptoms of PTSD as risk factors for increased impulsivity and risk-taking behavior (Ben-Zur and Zeidner, 2009; James et al., 2014). Furthermore, acute stress has been shown to modulate decision-making processes, increasing individuals’ risk taking behavior in a time-dependent manner (Bendahan et al., 2017). In animal research, the EPM measures exploratory drive and anxiety in a novel environment, which is potentially useful for new niche discovery (i.e., food resources and reproductive partners) but also dangerous (i.e., predators). An increase in the open arm time or entries could therefore also be interpreted as an increase in risk-taking exploration (Macrì et al., 2002; Cortese et al., 2010). While comprehensive proof of the role of *Fkbp5* in the ovBNST on stress-induced anxiolytic behavior is still lacking, our data support this notion.

In summary, we present the first characterization of *Fkbp5’*s role in the ovBNST on HPA axis function and anxiety-related behavior. Our findings suggest that here stress induction of *Fkbp5* may have a protective role, leading to decreased anxiety and suppression of future stress-induced HPA axis activation. Further in-depth investigations of the causality will help to understand the full extent of the underlying mechanisms and lead to a better understanding of ovBNST’s role in stress-induced anxiety disorders.

## Synthesis

Reviewing Editor: Laura Volpicelli-Daley, University of Alabama at Birmingham Decisions are customarily a result of the Reviewing Editor and the peer reviewers coming together and discussing their recommendations until a consensus is reached. When revisions are invited, a fact-based synthesis statement explaining their decision and outlining what is needed to prepare a revision will be listed below.

This study was initially submitted to J. Neuroscience and was recommended for publication in eNeuro. The reviews were favorable and the authors addressed all the comments for the J. Neuroscience reviews. It would be nice to see an image detailing expression of Fkbp5 message, effect of various treatments, etc in a larger (half-hemispherical) brain section, and then provide a magnified image of the ROI. The lower magnification images alone make the reader immediately question whether the effects are localized.

Can the authors please address why 21 days of CSDS where required, and whether mice were injured during CSDS, if not, how do the researchers know? What steps were taken to prevent injury?

## Author Response

eN-TNWR-0425-21X

Engelhardt et al, 2021

Response to reviewers

The significance statement is appropriate for a general audience. It would be helpful to have a brief definition of FKB51 in the significance statement and abstract. Also, I recommend removing the word “first” in “ “Here we provide a first characterization”. Since there are almost 500 articles on FKB51 and stress, it is possible that this is not the “first” analysis ever performed.

Response:

We agree and have adapted the significance statement and the abstract accordingly.

This study was initially submitted to J. Neuroscience and was recommended for publication in eNeuro. The reviews were favorable and the authors addressed all the comments for the J. Neuroscience reviews. It would be nice to see an image detailing expression of Fkbp5 message, effect of various treatments, etc in a larger (half-hemispherical) brain section, and then provide a magnified image of the ROI. The lower magnification images alone make the reader immediately question whether the effects are localized.

Response:

We agree that showing an overview of a brain hemisphere would be nice. However, as the images were obtained in dark field mode at 20x, we cannot provide a larger field of view. In a lower magnification the silver grains in the slides do not reflect the light anymore and the signal is lost. We can therefore not provide an overview image due to technical limitations. As we have clearly stated in the text that virus spread to adjacent brain regions is possible and also provided a scheme with the injection sites for each manipulation, we believe this issue is sufficiently addressed.

Can the authors please address why 21 days of CSDS where required, and whether mice were injured during CSDS, if not, how do the researchers know? What steps were taken to prevent injury?

Response:

The 21-day CSDS paradigm is a widely used variation of the original 10-day CSDS. Here the emphasis is on the chronicity of the stress exposure and the continuation of stress exposure during behavioral assessments in the 3rd week of the protocol. To prevent injuries, CD1 aggressors are only allowed to attack the test animals 2-3 times and biting is prevented by manual separation of the mice directly following an attack. Total defeat time is also reduced and the animals are separated after the successful attacks of the CD1 aggressor, independent of the time this has taken. Therefore, in contrast to the classical 10-day CSDS protocol, bite wounds are avoided. All animals are scored daily for possible wounds and injured animals would be excluded from the experiment. We have now added a more detailed description of this procedure to the Materials and Methods section.
